# On the Effect of Interferences on X-Band Radar Wave Measurements

**DOI:** 10.3390/s22103818

**Published:** 2022-05-18

**Authors:** Pavel Chernyshov, Katrin Hessner, Andrey Zavadsky, Yaron Toledo

**Affiliations:** 1 Helmholtz-Zentrum Hereon, 21502 Geesthacht, Germany; pavel.chernyshov@hereon.de; 2 School of Mechanical Engineering, Tel Aviv University, Tel Aviv 6997801, Israel; andrei.zav@technion.ac.il; 3 Ocean Waves GmbH, 21339 Luneburg, Germany; hessner@oceanwaves.de; 4 Coastal and Marine Engineering Research Institute (CAMERI), Haifa 3200003, Israel

**Keywords:** X-band radar, harbor area, radar image preprocessing

## Abstract

X-band radars are in growing use for various oceanographic purposes, providing spatial real-time information about sea state parameters, surface elevations, currents, and bathymetry. Therefore, it is very appealing to use such systems as operational aids to harbour management. In an installation of such a remote sensing system in Haifa Port, consistent radially aligned spikes of brightness randomly distributed with respect to azimuth were identified. These streak noise patterns were found to be interfering with the common approach of oceanographic analysis. Harbour areas are regularly frequented with additional electromagnetic transmissions from other ship and land-based radars, which may serve as a source of such interference. A new approach is proposed for the filtering of such undesirable interference patterns from the X-band radar images. It was verified with comparison to in-situ measurements of a nearby wave buoy. Regardless of the actual source of the corresponding pseudo-wave energy, it was found to be crucial to apply such filtration in order to improve the performance of the standard oceanographic parameter retrieval algorithm. This results in better estimation of the mean sea state parameters towards lower values of the significant wave height. For the commercial WaMoSII system this enhancement was clearly apparent in the improvement of the built-in quality control criteria marks. The developed prepossessing procedure improves the robustness of the directional spectra estimation practically eliminating pseudo-wave energy components. It also extends the system’s capability to measure storm events earlier on, a fact that is of high importance for harbour operational decision making.

## 1. Introduction

X-band nautical radars are popular sensors commonly used by ships and harbors for navigation and traffic management. Nevertheless, in recent years they are also widely used as a measurement and observation instrument of sea waves, currents, bathymetry, and environmental conditions. In the near-range (<4 nautical miles), marine radars receive backscatter signals from the sea surface, which become visible as sea clutter in the radar image. These signals are created by Bragg resonant interaction between the transmitted electromagnetic waves and the corresponding sea waves, which are ripples in the order of a few centimeters in wavelength. Surface waves such as wind sea and swell in the order of several 10’s of meters become visible since they modulate the sea clutter through tilt, shadowing, and hydrodynamic imaging mechanisms [1,2,3].

Shadowing, speckle noise, and tilt modulations are considered to be the main imaging mechanisms for the grazing incidence angle of probing which is typical for land-based radar applications [4]. In order to obtain the original sea elevations, a reconstruction procedure is required, which mainly aims to filter out speckle-noise, and higher harmonics resulting from the nonlinear shadowing mechanism by means of band-pass filtration in frequency, wave vector or frequency-wave vector domain (e.g., dispersion relation filter [2]).

To obtain wave spectra from intensity radar images, temporal sequences for their Cartesian sub-areas (I(r,t)) are transformed into the spectral domain by means of 3D Discrete Fast Fourier Transform (3D DFFT), which results in a 3D image spectrum $S^{3}\left[I\right]\left(k,\;\omega \right)$.

The unambiguous directional wave spectra are retrieved, extracting the wave related spectral energy from the $S^{3}\left(k,\;\omega \right)$ by means of the linear dispersion relation as band-pass filter function and subsequent application of a modulations transfer function (MTF) to compensate for nonlinear imaging effects such as shadowing and tilt modulations [5]. Finally, the corresponding filtered spectrum is calibrated to the actual value of the significant wave height which is either derived from the radar using Signal-to-Noise Ration (SNR)-based approach or using independent in-situ measurements. More details on the individual processing steps can be found in the literature (see e.g., [6,7,8] ).

In this paper, the marine radar-based oceanographic measurements were carried out using the sigma S6 WaMoSII system, which is a commercial off-the-shelf remote sensing system consisting of a high-speed video digitizing and storage device. The directional unambiguous sea-state information, the information of surface current ([9,10]), and, in shallow water, the water depth can be derived [2,11]. The standard WaMoSII software delivers unambiguous directional wave spectra and time series of the integrated spectral parameters such as: significant wave height ($H_{s}$), peak wave period ($T_{p}$), peak wave direction ($D_{p}$) describing the key properties of the sea-state in near-real time.

Various studies have proven the reliability and accuracy of wave observation by WaMoSII from offshore platforms, vessels and from coastal stations [3,12,13]. Recent studies of coastal WaMoSII installations have shown that the local weather conditions (rain, calm wind) may influence the quality of the WaMoSII measurements (see e.g., [14]). Therefore, WaMoSII includes a real-time quality control which indicates whether the resulting data passed specific Quality Control (QC) checks. For present application, the WaMoSII QC is of a special interest, since the Haifa Port is a challenging location with various stationary and moving objects within the radar footprint (ships, breakwaters, etc.) and nearby transmitting radars, which requires a proper identification of reliable sea state information.

For the rapidly changing nearshore environment, not only the QC application but the signal processing approach also needs to be adjusted. Due to refraction and shoaling processes in the shallower water depths ($k_{p}h<1$), wave signal and the corresponding sea clutter can not be considered as periodical and homogeneous processes, especially in the spatial domain. This issue is regularly addressed by using either windowed Fourier transform (see e.g., [15,16]) or Wavelet transform ([17,18,19]). Nevertheless, for this paper the conventional signal processing approach will still be performed, taking rectangular analysis areas in such a way that the water depth does not vary significantly inside of it.

The harbour nearshore environment is also challenging due to the following circumstances. This area is regularly frequented by ships, especially during storm events. It is also overfilled by undesirable radar–radar interference, since each boat, even a small one operates its own navigational X-band radar, in addition to the navigational radars for controlling the harbour traffic used by ports. Another type of undesirable interaction occurs with tall solid structures which provide sheltering of the actual sea surface with waves from the electromagnetic waves emitted by the radar.

Ports are responsible for informing inbound, outbound, and moored ships of impending inclement weather conditions usually based on the information from forecasts. However, weather/wave forecast conditions may deviate from actual weather conditions within a range of few hours, therefore observation of actual conditions and identifying trends of wave parameters’ variation is of a high importance for port operation.

**Figure 1:**
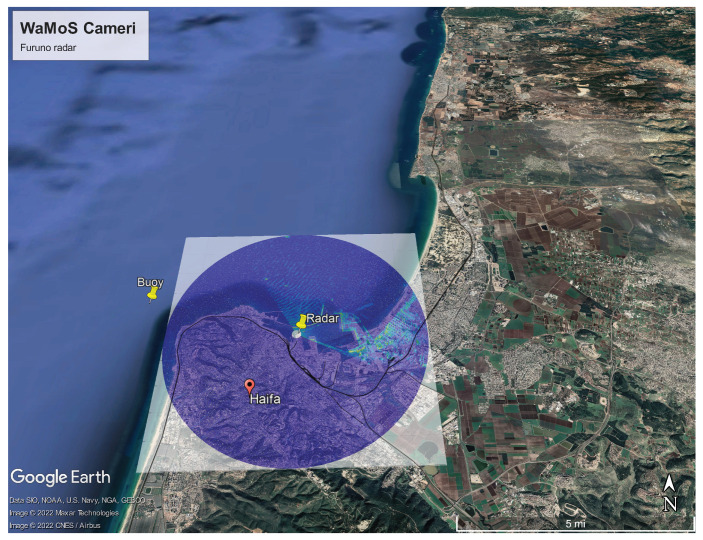
Google map showing the position of the WaMoSII radar system in Haifa port and the associated radar range of 5 km. The overlay radar image shows signatures of ships and breakwater as well as sea clutter. Furthermore the location of the reference buoy offshore is marked with a yellow pin near the southern cape of the bay.

Monitoring of the sea-state near Haifa Port is carried out using a Waverider buoy (see Figure 1). Although it is a highly reliable tool, it has several limitations. The buoy is located outside of the bay, therefore providing only approximate information on the sea state at the proximity of the port. The last is due to the fact that the waves approaching the area adjacent to Haifa port entrance undergo shoaling deformations and refraction, which cannot be neglected. Furthermore, the buoy data require integration time of approximately half an hour for providing the sea-state condition, which is an undesirable delay in operational decision making. Therefore, additional instruments, such as oceanographic X-band radars can be deployed as a part of an integrated system to provide more reliable spatio-temporal information in the vicinity of the port’s entrance.

Assuming there are sufficiently high waves in the view field of the radar, their visibility in marine X-band radar images and hence the quality and reliability of the wave observation depends on environmental conditions, e.g., wind, which is responsible for creating sea clutter. Also rain clutter or interference in the image can camouflage the actual wave patterns and hence can degrade the quality of the sea state estimations ([20,21,22,23]).

**Figure 2:**
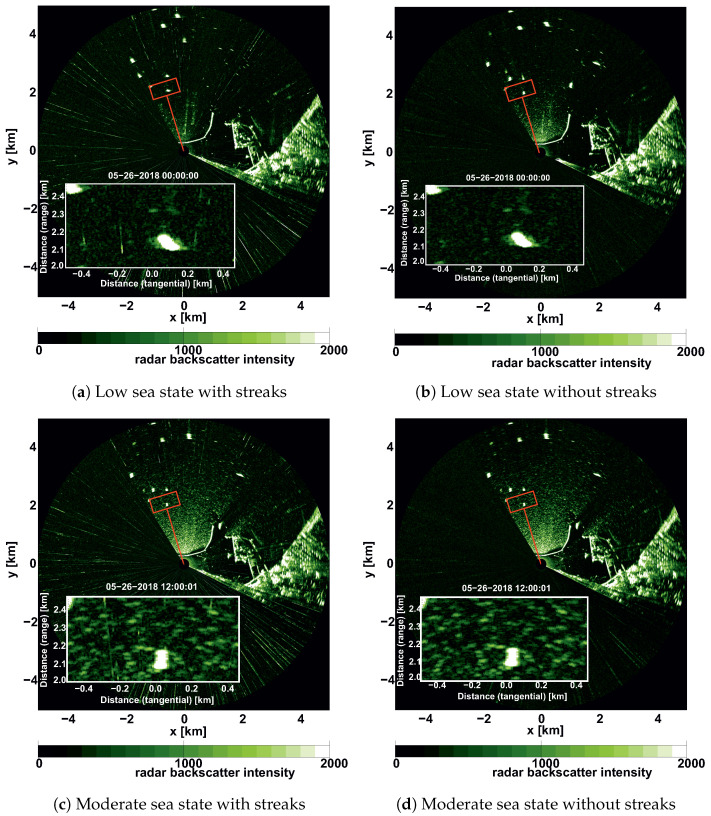
Examples of the WaMoSII X-Band radar images of a low ($H_{s}\approx 0.6$ m, wind speed 3.2 m/s) and moderate sea states ($H_{s}\approx 1$ m, wind speed 5.8 m/s), acquired at Haifa port given before (left column) and after (right column) streak filtration procedure application. Zoomed areas correspond to the analysis area of box 1 (red rectangle). Ships are clearly visible as a bright spots within the radar footprint.

Low and moderate sea states are characterized by comparatively weak sea clutter resulting in a low intensity of the wave signatures in comparison to background patterns. These patterns include combinations of speckle noise and interference with other radars which manifests as high energy streaks appearing on the radar image (see examples of the images in Figure 2). Since their level is relatively high with respect to signal, the SNR becomes low and the quality of the data is significantly reduced.

The integral amount of energy associated with such interference is still quite low since they are manifested just in several azimuthal directions not even affecting the whole range string. These patterns are not dispersive and appear to be random in time and space.

An important findings of this paper is that it seems to be the first time when the effect of streak patterns on the quality of the radar-based sea state parameters estimation is revealed. A motivating observation was that after standard WaMoSII wave parameters’ estimation a significant amount of false QC classification, especially during low sea states, was noticed. In some cases wave spectra, which seemed to be including valid sea state information were regarded as unreliable. In a handful of other cases, spectra with artefacts were regarded as reliable.

This finding led to more thorough analysis of the mentioned wave spectra and hence some pseudo-wave energy was detected (refer to the Section for details). These artefacts in physical domain were always aligned in radar look direction independently on the alignment of the analysis window area. The last could be expound by the hypothesis that these artefacts are correlated with the interference streaks which are aligned radially. To validate this hypothesis a streak filter which removes the interference signatures was implemented. After removing the streak signals from the radar images (as a prepossessing step), it turned out that the artefacts in the wave spectrum disappear, which proved the initial hypothesis. Furthermore, the filter led to a significantly better wave analysis performance in low and moderate sea state conditions as well as a more reliable QC assessment.

The paper is organized as follows: Section 2.1 described the oceanographic marine radar installation, characteristics, and geographical location and that of the supporting in-situ measurements, Section 3 includes the theoretical analysis of the streak spectrum depending on the probing and analysis area geometry as well as a general idea of the streaks’ spectrum filtration procedure, which will be applied to obtain the results summarized in Section 4. The results are shown in both aspects, such as broadening of the result on the timeseries comparisons radar with the corresponding in-situ buoy measurements, directional wave spectra, and on statistical analysis of the corresponding results’ histograms before and after filtration. The paper is finalized by a brief discussion and conclusions section giving an overview of the main results.

## 2. Materials and Methods

### 2.1. Description of the Wamosii Installation in Haifa Port and Accompanying In-Situ Measurements

Wave measurements near Haifa bay area have been carried out by the Coastal and Marine Engineering Institute (CAMERI) since November 1993 for the Israel Ports Development and Assets Company. For that purpose a Datawell Mk-III directional waverider spherical buoy was deployed near Haifa bay (32°50.5′ N and 34°56.2′ E) as shown in Figure 1. The water depth at the buoy location is approximately 24 m.

**Figure 3:**
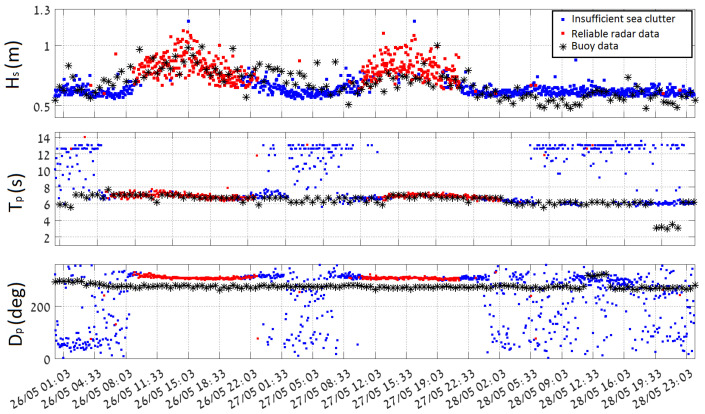
Comparison of the radar image sequence derived sea state parameters ($H_{s}$, $T_{p}$, $D_{p}$), calculated for the analysis box 2 (see the box definition in Figure 4) and the corresponding wave rider buoy measurements for the mention period of 26–29 May 2018 (times are given in UTC). Streak filtration procedure is not introduced to the preprocessing. Reliable data and insufficient sea clutter radar data are marked based on the inner WaMoSII quality control criteria.

The data are processed based on thirty-minute-long time series, acquired with 1.28 Hz sampling frequency on the buoy side. The wave spectra are computed by the corresponding spectral information averaging from the sequence of eight 200 s long windows without overlapping. At the end of each thirty minutes all mean spectral parameters are logged and become available for downloading and further applications. A comparison between the wave parameters derived from the radar image sequences and one obtained by the buoy is given in Figure 3.

The oceanographic radar station was installed by the CAMERI in cooperation with Marine Engineering and Physics Lab (MEPL) with the support of the Israeli Port Company (IPC) and is located at the Haifa Port (32.8222° N 35.0085° E) as shown in Figure 1. The station consists of the WaMoSII data acquisition and processing block connected to FURUNO navigational radar Far 2117 XN-24AF (the corresponding specifications are summarized in Table 1). WaMoSII with SIGMA S6 software allows carrying out real time acquisition and processing of individual radar images and performing further data analysis in order to extract the waves, currents, and bathymetry information.

In addition to the radar, a Gill WindSonic ultrasonic wind sensor was deployed and connected directly to the WaMoSII. During radar data acquisition the wind information is merged into the radar data. During the processing, the wind information allows identification of calm weather situations in which no radar-based wave and current estimations are possible.

**Figure 4:**
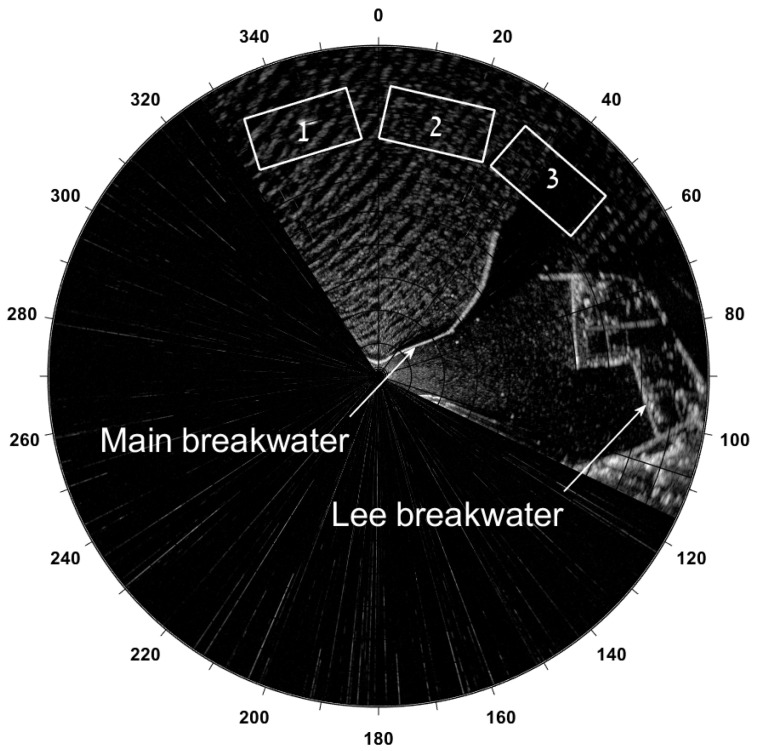
WaMoSII radar image acquired in Haifa bay, showing the main and lee breakwaters and location of the WaMoSII wave analysis areas at 2 km off the radar.

**Figure 5:**
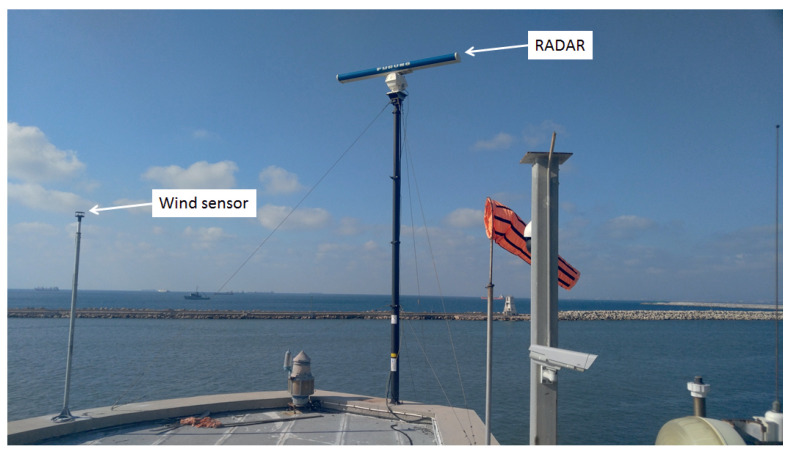
Photo of FURUNO navigational radar Far 2117 XN-24AF antenna connected to the WaMoSII system situated inside the control tower building of the Haifa port. Ultrasonic wind sensor installed as a support part for the WaMoSII processing system. In the far field, the main breakwater of the harbor is visible.

Both radar and wind sensor are mounted on the top of the extendable (telescopic) tower installed on top of the Haifa port’s control tower as shown in Figure 5. The radar installation height is about 18 m above mean sea level. The radar data sampling starts at a range of 100 m up to 5 km from the radar antenna. The radar footprint is limited to the azimuths of 328–117° relative to North in order to focus on the area of interest. Figure 4 shows an example of the WaMoSII radar image. For the Haifa radar station, 3 analysis areas were defined to compute the wave spectral parameters. The numbering of the analysis areas is given as 1–2–3 from left to right. The rectangles’ dimensions were chosen as 1000 m long and 500 m wide. This choice of the analysis areas’ dimensions was conditioned on one hand by the minimum number of wavelengths fitting the computational area and on the other hand, by keeping it small enough that the bathymetry variation would not affect the waves within the area. The center of each rectangle is located at the radial distance of 2 km from the radar and at the angles of 343°, 13° and 41° degrees relative to the North direction for boxes 1, 2 and 3 respectively. Further only the results from the box 2 are discussed. In Figure 2 also the only one analysis area is shown.

Since the location of the buoy differs from that of radar analysis boxes, and waves undergo a noticeable deformation due to refraction and shoaling, wave state parameters measured by the instruments cannot be compared directly. Hence these results can be considered to be only qualitative.

### 2.2. Radar Location and Challenges

Radar station location in Haifa port is challenging in several aspects for wave observations. First of all the analysis areas’ locations are sheltered by the cape, therefore waves are strongly refracted and the significant fraction of wave fronts run into the radar side-look direction.

Some area outside the breakwaters is shadowed due to both the size of the breakwater’s construction and comparatively low radar installation height. As a result, analysis box 3 is located almost entirely in the shadowed zone as appears in Figure 4.

Another issue is that during storm events a lot of ships are standing anchored within the radar footprint outside the port’s area (see Figure 2). Ships are visible as spots of localized high backscatter intensity. High intensity streaks are appearing as dash-dotted-like lines in random radial directions (refer to the left column of Figure 2). The interferences can come from other radar sources, e.g., stationary radars used by the port authorities, or other transmitting sources in the vicinity of the WaMoSII radar. Range intensity trend decay corresponding to the radar equation is also noticeable.

It is important to point out that the streaks contamination affects mostly low and moderate sea states. The intensity of the interferences does not vary with environmental conditions, but is affected more by the power of radar transmission, whilst the sea surface backscatter intensity depends on the wind, wave orbital velocities, and white capping intensity.

## 3. Methods

### 3.1. Streaks Spectrum

**Figure 6:**
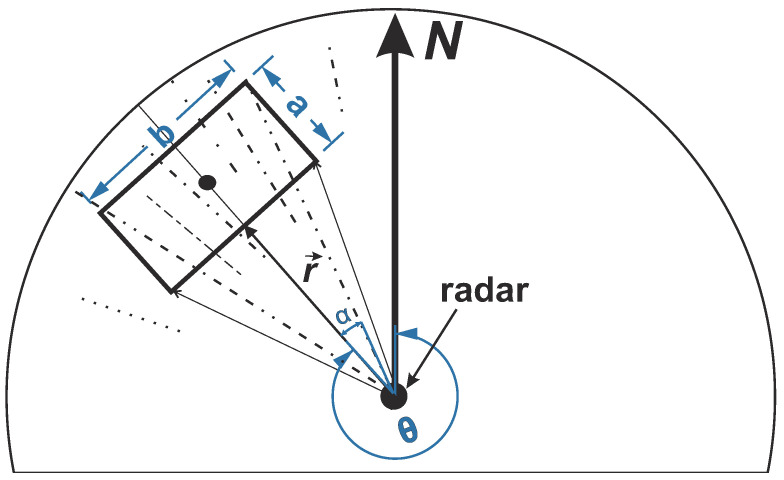
Geometry of the analysis box situation to understand the directional spikes component distribution in the spectral domain.

In this section the qualitative description of the streaks’ spectrum will be given. As it was already mentioned in the introduction, streaks are manifested on radar images as a radial spikes of high intensity (Figure 2a,c). This streaks are regularly one azimuthal pixel wide. Taking into account a Pulse Repetition Frequency (PRF) of 3kHz the actual (raw) azimuthal resolution is 0.12°, which before interpolation corresponds to around 5 m of an azimuthal resolution within the box. If one examines a typical geometry of the standard analysis box located within the radar image (Figure 6), the corresponding streak patterns behaviour looks similar to that of the laterally propagating quasi-periodical fronts without any defined dispersion law.

**Figure 7:**
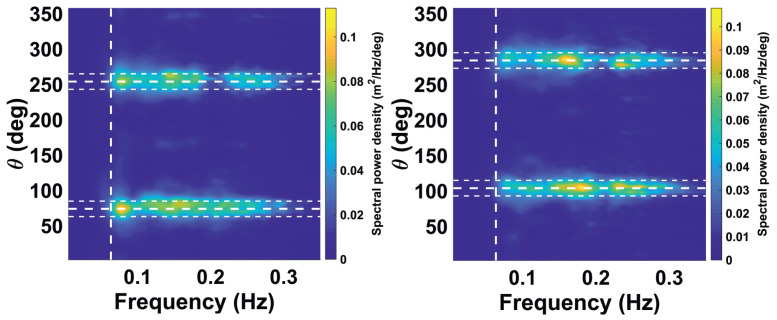
Streaks spectra examples from box 1 (344° N) (**left panel**) and box 2 (14° N) (**right panel**). For the positioning of the corresponding analysis boxes on an actual radar image, please refer to the Figure 4. Both spectra are measured at the calm sea state ($H_{s}\approx 0.6$ m) 27 May 2018 04:02 a.m. (refer to Figure 3).

Therefore streaks will distribute their energy in frequency-wave number spectral domain quite widely (not necessarily uniformly) within wave number limits $k_{s}\in \left(k_{0},\;k_{N}\right)=\left(2\pi /b,\;\pi /\Delta y\right)$, where *b* is a lateral width of the corresponding analysis window (see Figure 6), Δy is the lateral resolution after the polar to Cartesian transformation. Within the frequency domain, assuming the dispersion law ω=ω(k), just the following inequalities $\omega \left(k_{0}\right)-\Delta \omega <\omega_{s}<\omega_{N}=\pi /\Delta t$, where Δω is the half-width of the dispersion relation shell, Δt - is the antenna revolution period (refer to Table 1) might be induced. As for the directional properties it is quite easy to derive from the geometrical representation, that it will depend on the corresponding analysis box rotation angle with respect to the North direction (θ). So maximum of the energy will be concentrated in the sector (θ±π/2)±α, where α=arctan(b/(2(r+a))) is an angular half-width of the corresponding streak spectrum, *a* and *b* are the range and lateral box dimensions respectively. Hence in the case when the box size is fixed if it is closer to the radar, then broader is the corresponding streak spectrum, and vise versa. To observe the effect of the streak spectrum aligning perpendicular to the box orientation refer to the Figure 7.

### 3.2. General Idea of the Filtration Procedure

**Figure 8:**
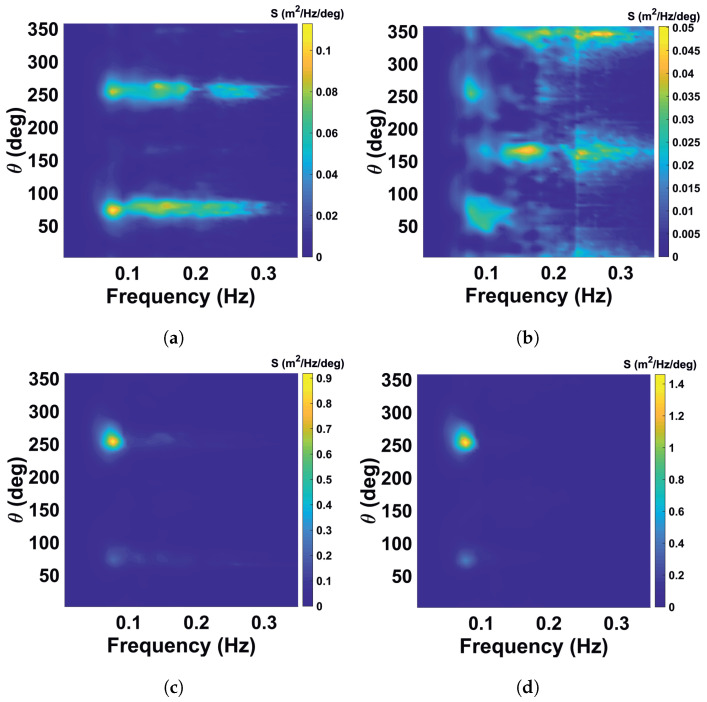
WaMoSII frequency spectra for insufficient sea clutter (upper row) marked as unreliable data and insufficient sea clutter marked as reliable signal (bottom row). The left column shows the standard results including artefact of the interferences. In right column the corresponding spectra after interference removal are shown. Streaks spectrum is well-pronounced in its theoretical location. (**a**) insufficient sea clutter (II) with artefacts before filtering. (**b**) insufficient sea clutter after streaks filtration. (**c**) insufficient sea clutter marked as reliable data (IR) with artefacts before filtering. (**d**) insufficient sea clutter marked as reliable data (IR) after streaks filtration.

**Figure 9:**
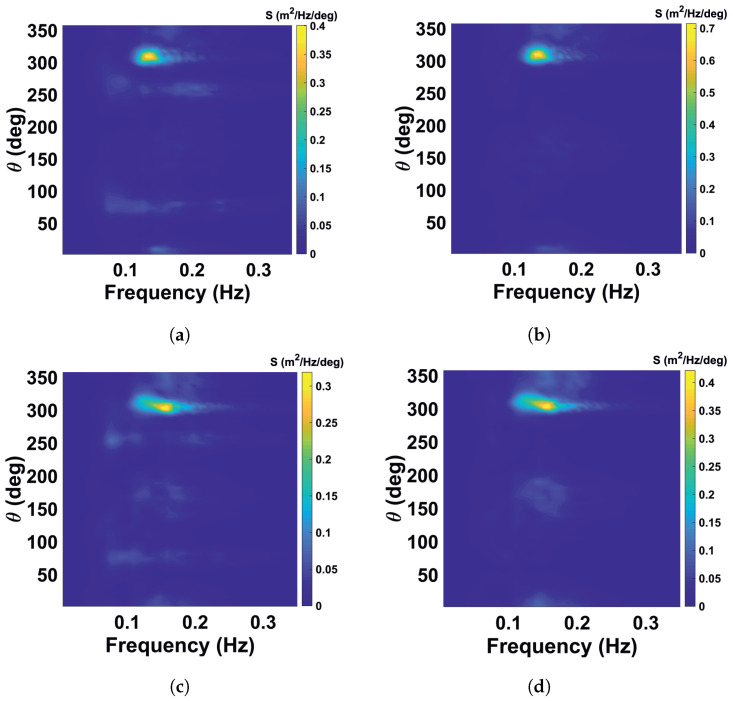
WaMoSII frequency spectra for reliable data marked as unreliable one (upper row) and reliable data (bottom row) before and after filtration. The left column shows the standard results including artefacts of the interferences. In right column the corresponding spectra after interference removal are shown. Streaks spectrum is still noticeable in its theoretical location. Right column panels demonstrate necessity of the streak filtration (weak streak patterns are efficiently removed). (**a**) Reliable data marked as one with insufficient sea clutter before filtering. (**b**) Reliable data marked as one with insufficient sea clutter after streaks’ filtration. (**c**) Reliable data marked correctly before filtration. (**d**) Reliable data marked correctly after streaks’ filtration.

Standard methods to mitigate radar interference like scan averaging are not suitable since they also suppress the variation of the sea clutter which is the carrier of the wave information. Therefore, the streaks are localized and removed individually, and the remaining gap is filled by interpolating the corresponding backscatter information between neighboring points. The analogous filter was previously applied as a preprocessing step for identification of rain and low-backscatter regions of X-band radar images in [22]. Some video denoising algorithms are given in [24]. The procedure is applied on individual radar images before the Cartesian transformation. Figure 2 shows the WaMoSII radar images before (left column) and after streak removal (right column) for the case with insufficient sea clutter (top, 26 May 2018, 0:00 UTC) and for the case with significant sea clutter (bottom, 26 May 2018, 12:00 UTC). In Figure 8 the corresponding frequency direction spectra are shown. On the left are the results with interference streaks. In the case of insufficient sea clutter (top left), the artefacts due to the streaks are visible. In the other case with sufficient sea clutter (bottom left), the energy of interferences is less than the wave signal’s energy, which dominates in the spectrum. When the streaks are removed from the radar images, the corresponding spectrum becomes a regular noise spectrum in case the insufficient sea clutter (top right). In the case with sufficient sea clutter (Figure 9), the filtering lead only to a minor differences in the area of the theoretical streaks’ location in the spectral domain.

## 4. Results

In this section the effect of the streaks filtration on the quality of sea state data will be outlined and summarized. For comparison, all the plots will be given in two versions: before and after filtration to visually estimate the impact.

Two breeze events, which took place during the time period from 01:03 UTC on 26 May 2018 to 23:03 UTC on 28 May 2018, when the significant wave height exceeded 1 m twice, were chosen for the comparison. The measurements relate to waves with period of 6–7 s. The waves main propagation direction is from N-W. Inside the radar footprint, the wavefield undergoes refraction and shoaling due to changes in bathymetry (see $D_{p}$ (lower) panel of Figure 3).

In Figure 3, the time series of the radar derived sea state parameters and the corresponding buoy data (black stars) are shown. The red and blue colours refer to data sets which either passed or did not pass the internal WaMoSII QC respectively. Passing the QC indicates that the results are reliable (see [14]). This was the case during the periods with sufficient wind speed (>3 m/s) and wave heights (>0.7 m). During the periods with insufficient sea clutter (blue) the corresponding QC classified the data to be unreliable. In those cases, the direct WaMoSII wave measurements ($T_{p}$ and $D_{p}$) exhibit unrealistically strong variation over the measurement range. It is important to notice, that the measurement area was chosen in a range of 2–3 km, which is a relatively far range for WaMoSII application and the expected sea clutter is weaker for this distance (standard working range of WaMoSII is 500–1500 m). Hence, the received sea clutter power is even weaker relative to interference, which is almost independent of the range.

While inspecting the WaMoSII results more thoroughly, it turned out that the QC works in most of the cases correctly and identifies reliable data as good (IQ=0) and marks unreliable data with IQ>0. Nevertheless, in transient times between interval of insufficient and sufficient sea clutter there were some cases of weak sea clutter when QC failed. Both cases, reliable data marked with IQ>0 and unreliable data with IQ=0 take place. In Table 2 examples for false classification are given. While inspecting the falsely defined datasets it turned out that significant energy is accumulated in radar look direction. In cases of insufficient sea clutter, these energy peaks are identified as spectral peaks which the QC regards as reliable wave systems. In cases of weak but sufficient sea clutter an additional energy peak in radar look direction leads to a noisy spectrum and QC identifies these data set as unreliable.

**Figure 10:**
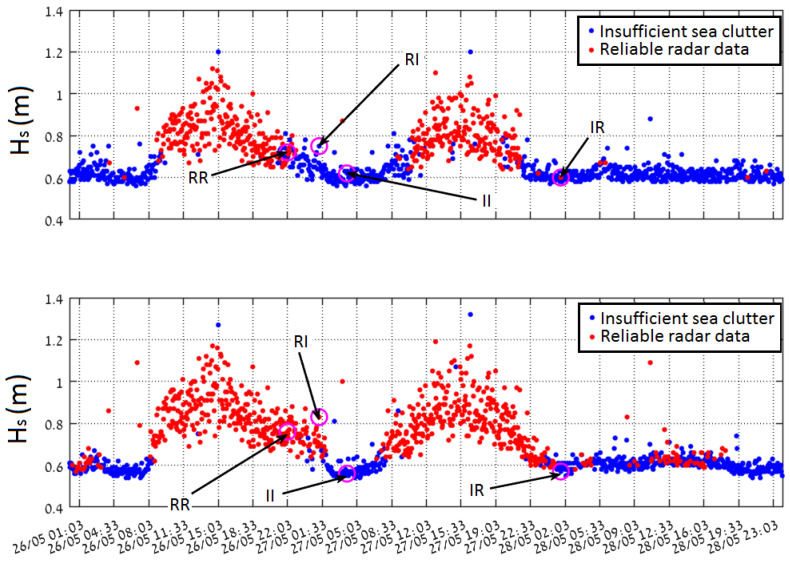
Comparison of the radar image sequence derived $H_{s}$, calculated for the analysis box 2 and the corresponding wave rider buoy measurements for the mentioned period of the storm event before (**upper panel**) and after (**lower panel**) the streak filtration procedure. All the special cases, such as (II), (IR), (RI), and (RR) are marked in the time series (for the description of the cases refer also to the Table 2).

If one compares $H_{s}$ time series before and after filtration (Figure 10) it is easy to notice that the duration of the high quality (reliable) data obtained from the filtered images is longer by about 3 h on each side of the local maximum value. The enhancement takes place mostly for the values of significant wave heights within a range of 0.5–0.7 m.

Four noticeable cases were chosen within the time series of the radar parameters to analyze the effect of filtration on their spectra. Cases are denoted as “insufficient sea clutter” and “reliable data” for our purpose.

The examples of above cases are indicated in Figure 10 and are also summarized in Table 2.

1. Noisy spectrum for very low sea state demonstrates pronounced streak spectrum pattern and after filtration shows normal noisy wave spectrum pattern (briefly is called as “insufficient sea clutter marked as an insufficient one” (II), see Figure 8a,b).
2. Some narrow lateral artificial features appear as regular wave spectra due to the superposition with streak spectrum (briefly is called as “insufficient sea clutter marked as reliable data” (IR), see Figure 8c,d). After streaks filtration the corresponding case does not pass QC criteria.
3. Normal wind wave (WW) sea spectral component due to presence of the superposed streak spectrum is defined as low quality by the WaMoSII quality control system (briefly is called as “reliable data marked as insufficient sea clutter” (RI), see Figure 9a,b) After streak filtration it is left as a normal WW component.
4. Wind wave component was not filtered out with the quality control criteria, but after filtration an artificial low frequency component associated with streaks is filtered out, so the resulting spectrum looks to be more relevant (briefly is called as “reliable data marked correctly” (RR), see Figure 9c,d).

The nature of the streaks noise phenomenon can be also understood by analyzing corresponding calibrated directional frequency power spectra S(f,θ). This spectral analysis approves theoretical qualitative description of the streaks spectrum given in Section 3.1 and shows superposition of the noisy, artificial and Wind Waves’ spectra with it. In general this analysis justifies the assumption of the crucial necessity of the streaks filtration procedure. It allows for elimination of streak patterns from the list of present spectral artifacts.

**Figure 11:**
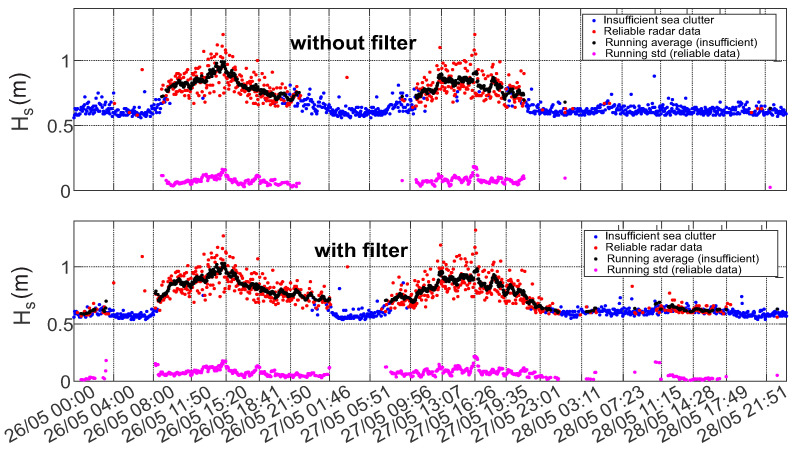
Time series of the significant wave height before (**upper panel**) and after (**lower panel**) streak filtration given together the moving average and std with the 20 min window size.

**Figure 12:**
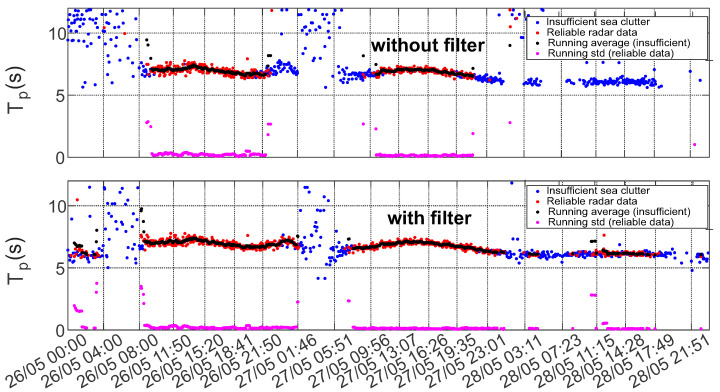
Time series of the peak period before (**upper panel**) and after (**lower panel**) streak filtration given together the moving average and std, calculated with the 20 min window size.

**Figure 13:**
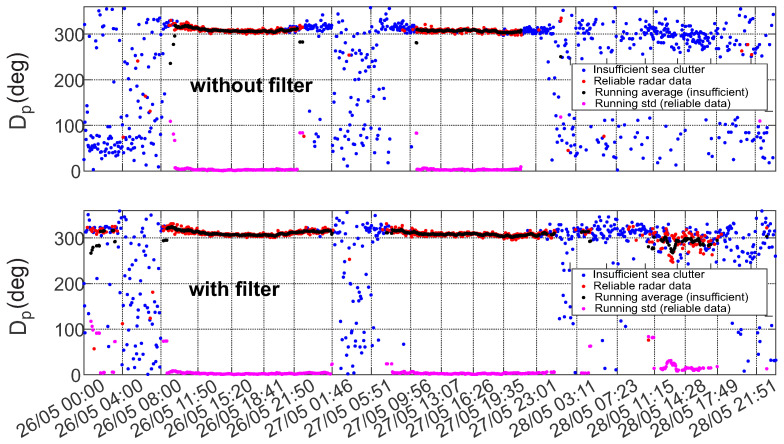
Time series of the peak direction before (**upper panel**) and after (**lower panel**) streak filtration given together the moving average and std, calculated with the 20 min window size.

The improvement of the results after removing the streak signatures also reflected in statistical analysis of the data (especially in low sea state conditions). This becomes apparent in the time series of the statistical sea state parameter (see Figure 11, Figure 12 and Figure 13). Figures show the comparison of time series of significant wave height, wave peak period and peak direction before and after the filtering, together with the moving average and moving standard deviation values of the reliable data. To obtain these values a 20 min wide window was moved through the reliable data with 2.5 min step. Within the window the mean and the standard deviation of the data was also calculated. The amount of reliable data increase and cover a wider range. Furthermore, the QC classification of the results shows better performance.

From the time series of these mean spectral parameters it can be seen that the effect of the streak removal is most significant during low sea state conditions, when the wave signal intensity is comparable to the inferences’ signals. This can be explained by the ability of the algorithm to process radar images with lower sea clutter intensity, which were discarded by QC before the filtering procedure was applied. The data scatter indicated by the standard deviation of the good quality data is not much affected.

**Figure 14:**
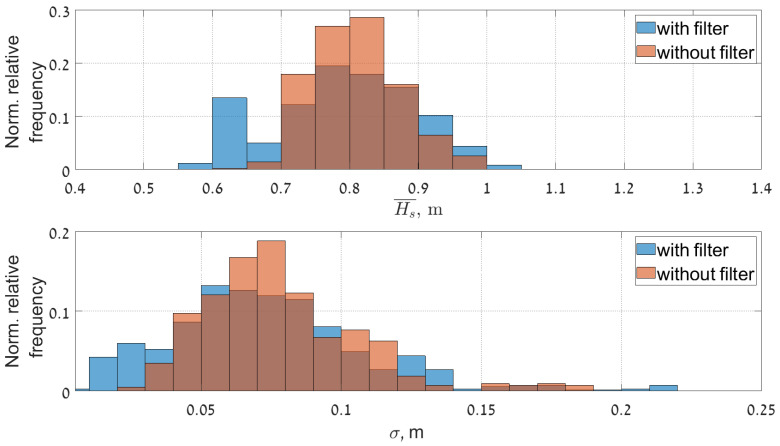
Histograms of the mean values of the $H_{s}$ and its standard deviation given in a relative fraction to the total number of points, bin width for mean value is 0.05 m and for standard deviation 0.01 m.

**Figure 15:**
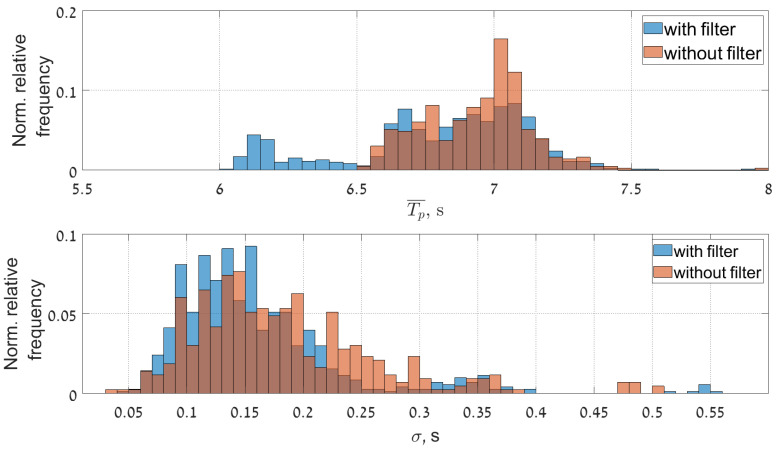
Normalized histograms of the mean values of the $T_{p}$ and its standard deviation given in a relative fraction to the total number of points, bin width for mean value is 0.05 s and for standard deviation 0.01 s.

**Figure 16:**
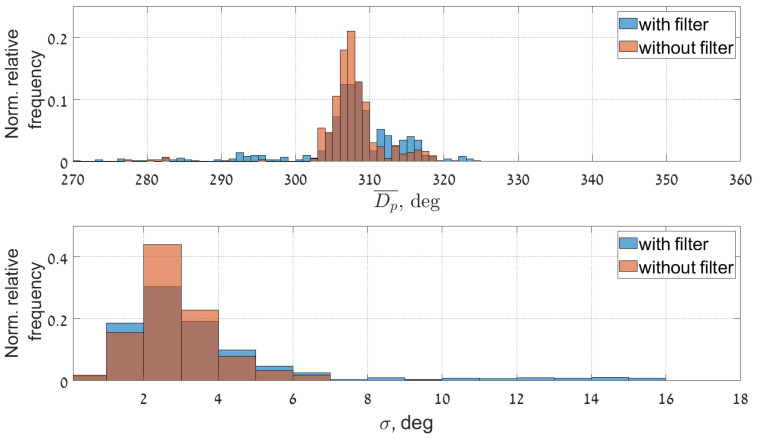
Normalized histograms of the mean values of the $D_{p}$ and its standard deviation given in a relative fraction to the total number of points, bin width for mean value is 1 deg and for standard deviation 1 deg.

Figure 14, Figure 15 and Figure 16 present normalized histograms of the corresponding moving average and standard deviation values. The histograms of the mean values of significant wave height in Figure 14 and the peak period in Figure 12 occupy wider range with respect to corresponding parameters for the cases when the filtering was applied, mostly for the lower values of significant wave heights (0.55–0.7 m) corresponding to the measured wave periods of 6–6.5 s.

The standard deviation of the wave height is slightly reduced and brought within the range of 0.01–0.15 m. The standard deviation of the wave period decreased slightly and after filtering exceeds somewhat 0.2 s.

## 5. Discussion and Conclusions

In the course of the experimental study carried out using oceanographic X-band radar in the vicinity of Haifa port, strong streak noise patterns were observed and identified to be interfering with the oceanographic parameter acquisition. Streaks noise can be considered to have an additive nature unlike the multiplicative speckle-noise effect, depending mostly on the configuration and the transmitted powers of the interfering sources.

This paper presents for the first time, that the signatures in radar images associated with interference can affect wave measurements. Since this interference appears to be random and sporadic in time and space it may have been formerly assumed that they do not affect the wave parameters’ estimations. This may be true in the open ocean or remote coastal stations, where interference signal plays a minor role. But for a port application such as Haifa harbor, this interference contributes significant energy, especially when the sea clutter is weak. This work showed that under such conditions such interference can indeed significantly affect the measurement, creating artefacts (pseudo-wave systems) in the wave spectrum which disturb the QC classification.

This type of the noise has different characteristics, which allows its filtration before Cartesian transformation in contrast to speckle-noise or rain clutter which are regularly filtered using different techniques in the Fourier spectral domain. The first motivation to introduce the filtration procedure was to justify that this strike interference is the cause of the artefacts in the spectrum. This was indeed confirmed. Finally, the following two positive effects of the interference removal were demonstrated:

1. It turned out that removal of the interference signatures leads to a significant improvement of the wave measurements, especially at low sea states. This improvement leads to higher coverage of reliable measurements on a wider range of sea states. In particular, it was shown that the method broadens its capabilities in the identification of lower and shorter waves.
2. The standard QC classification was more reliable, and false classifications were minimized such that their level was in the same range as for open ocean applications.

This filtration procedure is highly recommended for integration in any X-band radar image processing tool in the nearshore or offshore radar-frequented environment.

## Figures and Tables

**Table 1:** Radar characteristics (signal, acquisition, installation).

Parameter	Value
Carrier frequency	9.41 (GHz)
Signal polarization	HH
Pulse Repetition Frequency (PRF)	3 (kHz)
Antenna revolution period	2.5 (s)
Antenna length	2.5 (m)
Azimuthal resolution	0.95°
Spacing in range	4.5 (m)
Radar installation height	18 (m)

**Table 2:** Time stamps of the cases chosen by different quality control scores (IQ).

Case Name	Date, Time	IQ w/o Filter	IQ w/Filter
II	27 May 2018 04:02:16	600	600
IR	28 May 2018 01:36:55	0	600
RI	27 May 2018 01:14:16	600	0
RR	26 May 2018 22:02:39	0	0
